# Supplementary material for: Characterization of a Treponema denticola ATCC 35405 mutant strain with mutation accumulation, including a lack of phage-derived genes
Source: PLoS One. 2022 Jun 24;17(6):e0270198. doi: 10.1371/journal.pone.0270198 (PMC9231711; doi:10.1371/journal.pone.0270198)
Supplement: S1 Raw image — (PDF) [file pone.0270198.s001.pdf]

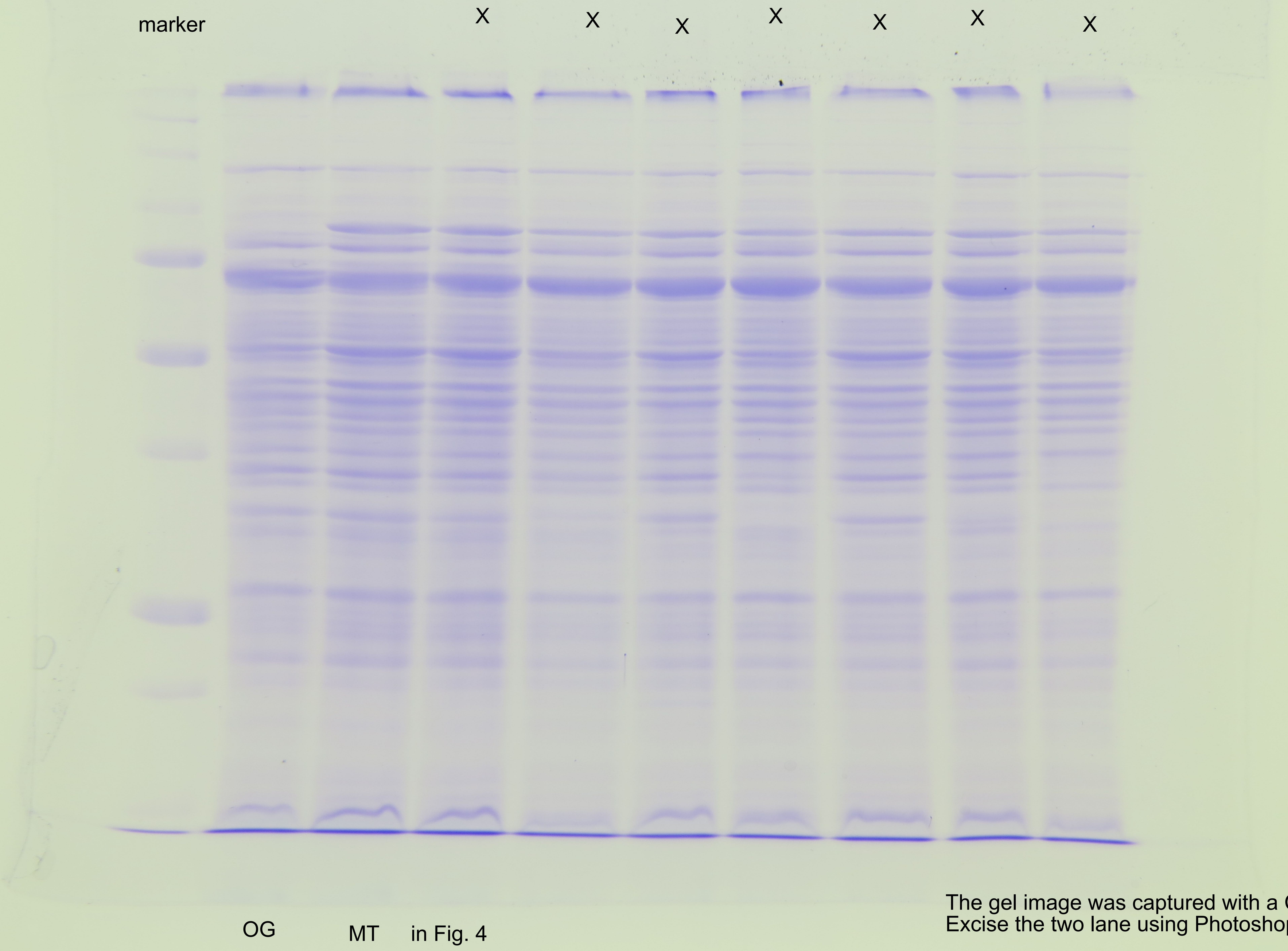

The gel image was captured with a CCD camera
Excise the two lane using Photoshop
