# Supplementary material for: Characterization of a Treponema denticola ATCC 35405 mutant strain with mutation accumulation, including a lack of phage-derived genes
Source: PLoS One. 2022 Jun 24;17(6):e0270198. doi: 10.1371/journal.pone.0270198 (PMC9231711; doi:10.1371/journal.pone.0270198)
Supplement: S2 File — DNA sequences around TDE_1173 and TDE_1133 of the original strain (ATCC 35405), a region detected in the MT strain (remaining sequence), the putative att sequence, and another repeat sequence. (DOCX) [file pone.0270198.s003.docx]

*Treponema denticola* ATCC 35405, complete genome

GenBank: AE017226.1

TDE_1174

TDE_1173

TDE_1133

Sequences surrounded with square: detected in the MT genome

AAGCCCTATTGCCGCTA: *attB*/*attP* predicted by Mitchell *et al*.(doi: 10.1099/mic.0.033654-0)

TTTAAAGGCACTCAAAWGAGTGCCTTTTTTTA: almost the identical sequence

AAGCCTTAAAAAAAGGCCCTATAGATATATTTGATAATGTATACCTCGGTGAAACAAGACCGCATTCTCTTATCCTTTGTATAGATGTACGCGGCTTTAGCGATTTTATGTGTAATAACGAAGAAACGGTTGTTTTCGGTCTAATTAAATCTTTTACATCCAACTTCCTTTCCTGCCTTAATCAGTTTGGTTATAATTGTTCTTATTATAAACTGCTTGGAGACGGAGCCTTAGTTATATGGGATAAGCTGGATGGGGTTGCAATAAAAGAAGCCGTTACGGTTTTTACTACATATGTAGAATTTACAAGAGAAGAACTATTTAAACCCTACGGAAATCTGGCAATAGGAGGTGCTTTAGTCCTAGATAAGGTATACAAATATGAAATTTCGGCAGAAGCATCTCAATTAAAATATAGAGATTATGTAGGTTACGGCATCAATCTTGCATGCCGCCTTCAAAACCTTGCAGCAGGCGGAGAACTTATCGTAAGTAAAAAATTGGTTGACATCGGTGCAATATATGCAACAAAAAATACAAACCCTGAAGTAATGAAAAAACTCAGAGCCCTTAAAGGAGTTAAACCTGAAGACAGGGAAGCTATCTTTTTGTATAAGGATATAAATCCGAAAATTATTTCGATATTTAAAGTTTTAAGCTTAGATTTCTAAATTAGGACTTGACATAAATGTATATTTTTTATATACTCAAAGCTCTTACGGCGGTATAGCTCAGTTGGCAGAGCAAACGGCTCATATCCGTTAGGTCATAGGTTCAAGCCCTATTGCCGCTAATCTTTTTTAAAGGCACTCAAATGAGTGCCTTTTTTTATTGTTTGTTTCACATCTTTATAGCTATTATAGACTTACACGATTATCACGTAAAGCACGATTTAACATTATTTTAGTTGTTTCTACTTTATTTTGCTTTATTTTTGCCGATATTGTAAGCAGTTTGTAAACTATATGTAAGCTATGAAACTTACTAAAGTGAGGGAAATATGAGCGTAAAACTATGGATTAACCGCAATAAAATATATCTTAGTATATATATCAGCGGTAAACGTTGGAGGGAAAGCACAGGGCTTACGGTTACTACCGATAAGGCTCAAAATAAGGTAGTTATGGATATGGCAGAAGTTTTGAGGAGCAAAAGGGAAGTTTCATTGATTGCCGTAAGTAACGGCTTATCAGATCCTGCTCTTACCAAAATAACCGTCCTTGAATATGTAAAAAATGCGGCTGCAGAAAAAAATAAAAAGCACCCGCTTCATAAGGTTGTTCTCTGGATTGAAAAAATATCGCCAACCTTAAAAATGGATGCTTTAACGCCGACATGGTTTGAAAACTTCCAGCAGACACTACAAAGAGAAACGGAGCTATCGCCTTATACTTGCGAAAATTACGCCTGCTCTTTACGTACTCTTTTTAAAAAGGCCGTAAGAGACGGAGTGCTGGTTAAAGATCCTACAATAGGAGTTAAACACATTCATTGCCCTGAAAGCATTAAAGAATTCTTAATGCCGGAAGATATTAGAAAATTGGCTGCAGAACCTATAGGCGGGATTTTAGGGGCGGATGTAAAAAAAGCCTTTTTGTTTGCATGCTGTACGGGCCTACGAATAAGCGACTTAAAAAGCTTAAAATGGGGCGGCGTATCTTTTGAGAAAAAGACACTTACAAAGATTCAACAGAAAACAAAGAGGGCCGTATATTTGCCTATCAAAGATGAGGCTATAGCTTTTTTGCACCTTCTTGCAGAGGAAAACCCGAACAGAACGGATGAAGATTTTATTTTTCCTCATGTTGCAACGACCGGCACCAATATGAATCAGTATTTAATAGAATGGGGCAAGCGGGCCGGAGTCAGACAGAAGATAGGCTGGCACTTGGCAAGACATACCCACGCTACCCTGCTTTTGGAGTCCGGGGCGGACTTATATACGGTTCAAAAACTTTTAGGCCATACCAAGATAAGCACCACGGCACAATATACACAAGTTACGGACAGGAAGAAAAAAGAGGCTATAGATTTGCTGCCTGATTACGGGATTGTGGGGGATTAG

ATGAAAAATAAGTTTATGTTTTTTGTGATTGTCGGGCTTGTAATTCTTTCGATTTTTATCGTGAATTACGGCCAGTCAATTACAACAGACTGCATCGGAGCAGTCATCGGCTTAGCGGGGGGAGCCTTAGCCGTGTTTGACACCGTCAAATACTCTCGGCGTAAGATTGCCCTTGTACCGCTATGGATTGGGATTTTGATTTTAATAGTTACCGGGTTTATACAGTTCAAAGGTGTTATTATTCTTGCAGCTTTCGGGTTTGCTGCCTTGGGACTGTATATATATTTTAGACTAACAGAAAAATAAAGTTTTTAATGTAAGGGGATGTTTATTTTAAGCAGCCCCTTTATTTATGTAAGCACTATGTAAGCAAGTATATTTATAATTTTATAAGTATATATTGAATATAGAATTATAAATCCGTAAATTAGATAAGCCCTATTGCCGCTAGCTAAATTTAAAGGCACTCAAACGAGTGCCTTTTTTTGTTTCCTAATTTTTAAAAACGCTAAAATCTTTCTATTTTTGAGACCAGAAAAGCATCCGCAAGAACTAAATAACACATTGCTTCCACAACCGGGACTATGCGAGGGAAAAGACAGATGTCGTGATTTCCTCCGACAGAAAGCATACATTTCTCCCCTTTTTTGTTAAAGGAGGCTTGGTTCATTTTTATTGAAGGAACGGGCTTTACGGCAATTCTAAAATCTATTTGACAAGTGTTCTCATCTTTTCTTCCATTCTCATGGTTTAAATTTAAGGGATAGTCCATATTACAGGAAATACCGCCCAAAATACCGCCAGAAAAATTTTTTGAAATGTCATTATTCTCGCTGCCTGTAATAGAAGCCGAGTAAAAACCGCCCCCGATTTCTATGCCCTTAACGGCACCTATCGACATTAAGGCTTGAGACAAGACCGCATCAAGTTTGCCGAAAACAGGAGAGCCGAGCCCTTCGGAAACATTTAAAACAGAACAGGATAAAATACAACCGGCCGAATCTCCATTAGAAGCCAAAGCGCTCAACTTTTCAAAAATCGGCTCAGGCAGAGCCTCATCTTCTTTAAGAGGGAGGCTTGTTTTAATACCTGCAATTTCTTCGGCTCTTACCTGCACCTCAATAGTTTTTTTACCGTCCTTTATAGCAAAGGCTTCGAGCATTTTTTTTGCAACAGCTCCGCCGATGAGCCTGCCTATCGTTTCCCGGCCGGAAGAGCGGCCTCCGCCTCGGTAATCCCGATGCCCGTATTTTAAATCATAAGAATAATCGGCATGCCCCGGACGATAAACATCTTTTAAGTTTTC
