# Supplementary figures and images for: Characterization of a Treponema denticola ATCC 35405 mutant strain with mutation accumulation, including a lack of phage-derived genes
Source: PLoS One. 2022 Jun 24;17(6):e0270198. doi: 10.1371/journal.pone.0270198 (PMC9231711; doi:10.1371/journal.pone.0270198)

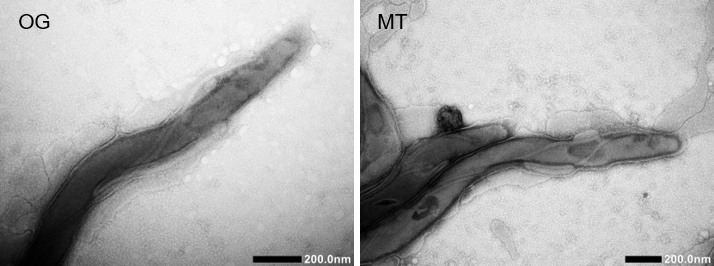

Supplement: S1 Fig — Two flagellar filaments were transparently observed from the end of the cells in both OG and MT. (TIF) [file pone.0270198.s005.tif]
